# Supplementary material for: Prediction of femoral osteoporosis using machine-learning analysis with radiomics features and abdomen-pelvic CT: A retrospective single center preliminary study
Source: PLoS One. 2021 Mar 4;16(3):e0247330. doi: 10.1371/journal.pone.0247330 (PMC7932154; doi:10.1371/journal.pone.0247330)
Supplement: S1 Table — (DOCX) [file pone.0247330.s001.docx]

|  | Fold 1 | Fold 2 | Fold 3 | Fold 4 | Fold 5 |
| --- | --- | --- | --- | --- | --- |
| Accuracy | 0.914 | 0.900 | 0.887 | 0.943 | 0.929 |
| 95% confidence Interval | 0.823–0.968 | 0.805-0.959 | 0.790-0.950 | 0.860-0.984 | 0.841-0.976 |
| P-value | 0.008 | 0.009 | 0.024 | 0.001 | 0.001 |
| Sensitivity | 0.714 | 0.600 | 0.733 | 0.786 | 0.867 |
| Specificity | 0.964 | 0.982 | 0.929 | 0.982 | 0.946 |
| Positive predictive value | 0.833 | 0.900 | 0.733 | 0.917 | 0.813 |
| Negative predictive value | 0.931 | 0.900 | 0.929 | 0.948 | 0.963 |
| Prevalence | 0.200 | 0.214 | 0.211 | 0.200 | 0.214 |
| Detection rate | 0.143 | 0.129 | 0.155 | 0.157 | 0.186 |
| Detection prevalence | 0.171 | 0.143 | 0.211 | 0.171 | 0.229 |

**S1 Table. Table of five-fold cross validation results of random forest model.**
