# Supplementary material for: Prediction of femoral osteoporosis using machine-learning analysis with radiomics features and abdomen-pelvic CT: A retrospective single center preliminary study
Source: PLoS One. 2021 Mar 4;16(3):e0247330. doi: 10.1371/journal.pone.0247330 (PMC7932154; doi:10.1371/journal.pone.0247330)
Supplement: S1 Appendix — (DOCX) [file pone.0247330.s002.docx]

**A graphical Summary of Random Forest Model**

#
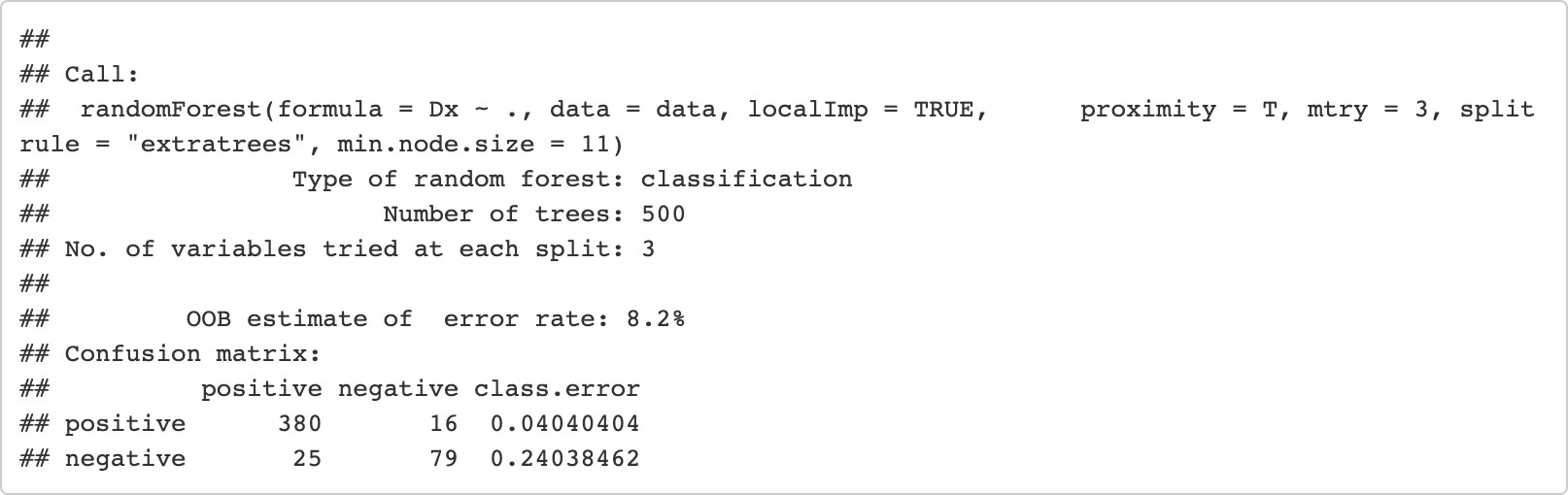
Details of random forest.

1. Importance measure of top 10 features.


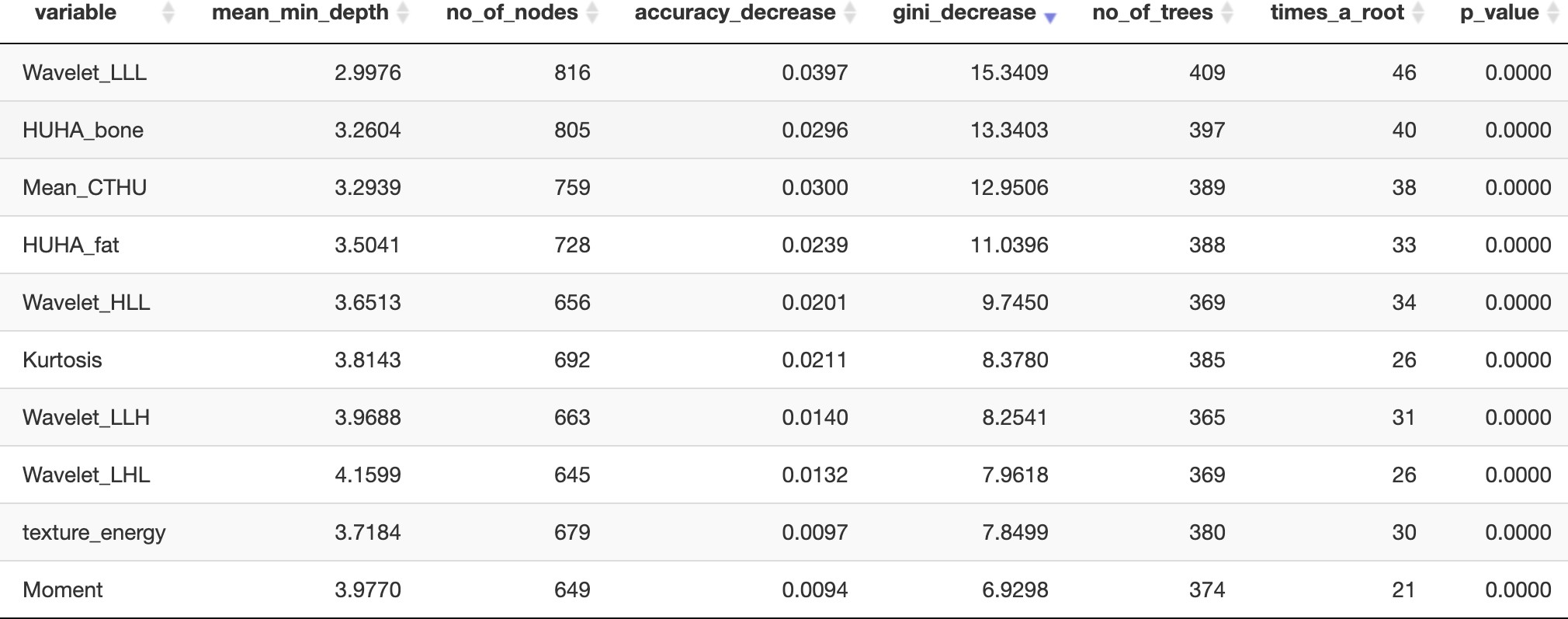


1. Variable interaction related conditional minimal depth.


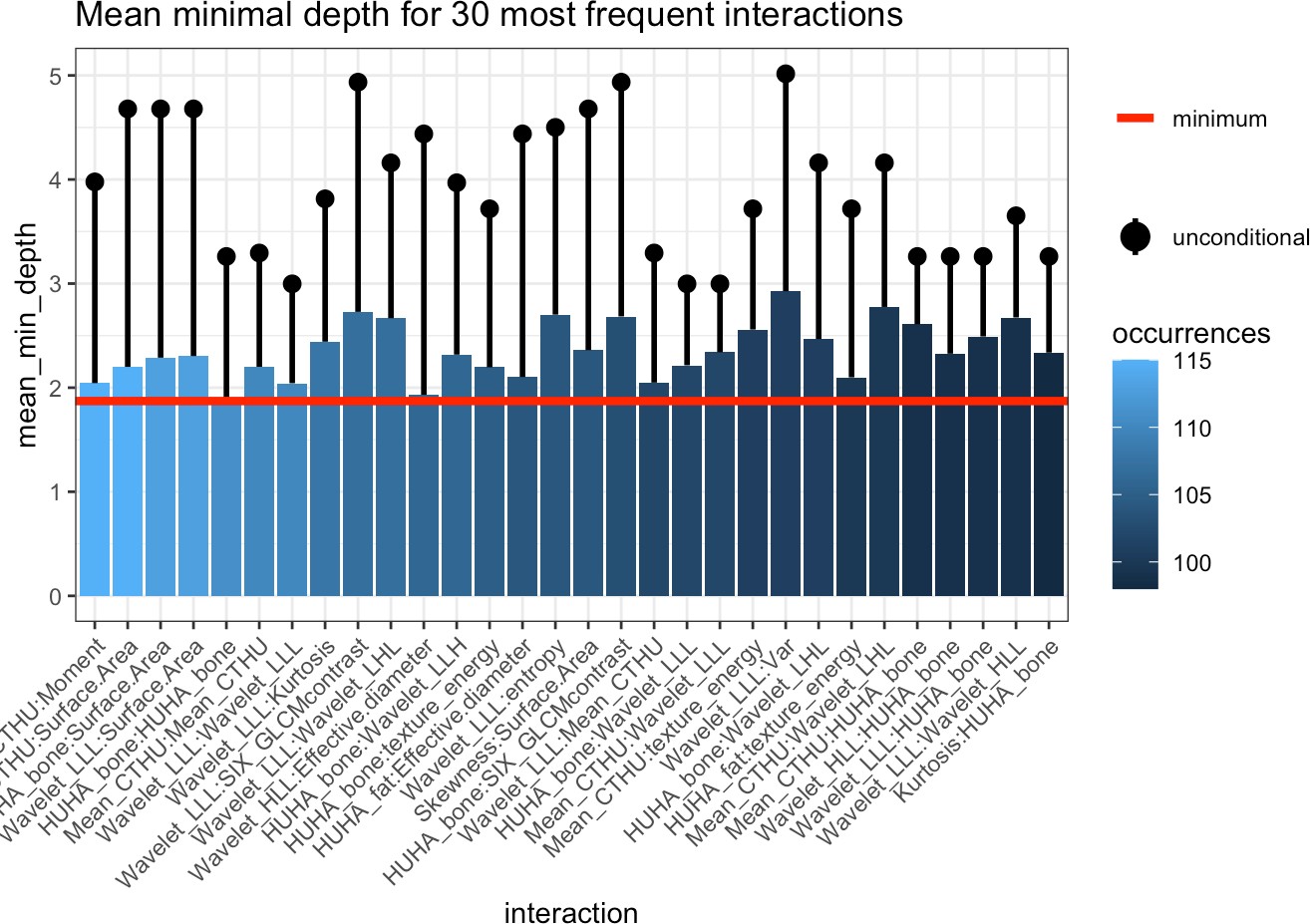


The plot above shows 30 top interactions according to mean of conditional minimal depth- a generalization of minimal depth that measures the depth of the second variable in a tree of which the first variable is a root (a subtree of a tree from the forest). In order to be comparable to normal depth 1 is subtracted so that 0 is the minimum. The horizontal line shows the minimal value of the depicted statistic among interactions for which it was calculated. The interactions considered are ones with the following variables as first (root variables): Wavelet_LLL, HUHA_bone, Mean_CTHU, HUHA_fat, Kurtosis, Wavelet_HLL, Wavelet_LLH, texture_energy, Wavelet_LHL, Moment, Skewness, entropy, Effective.diameter, Var, Surface.Area and all possible values of the second variable.

# Multiway importance plot.


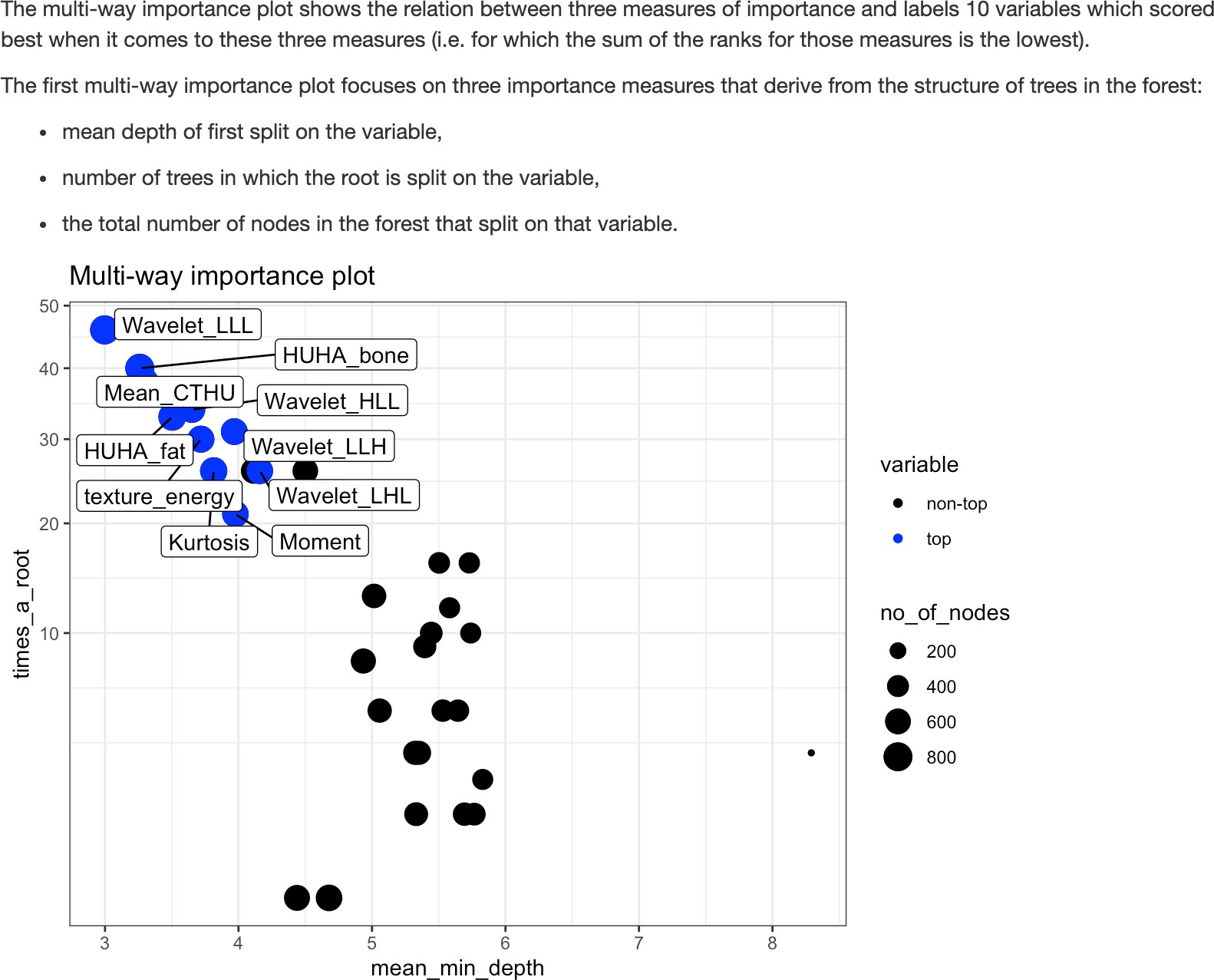


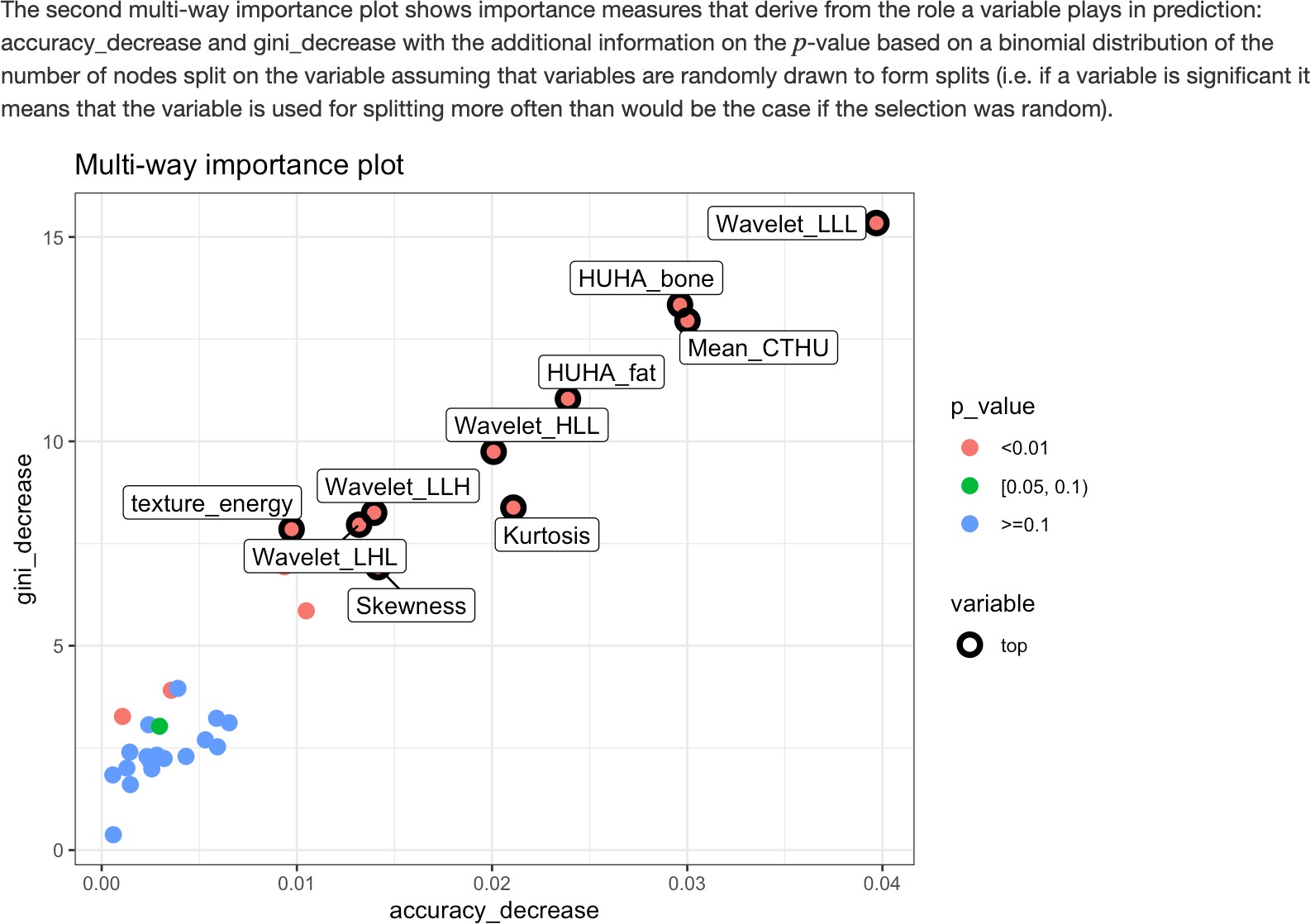


1.
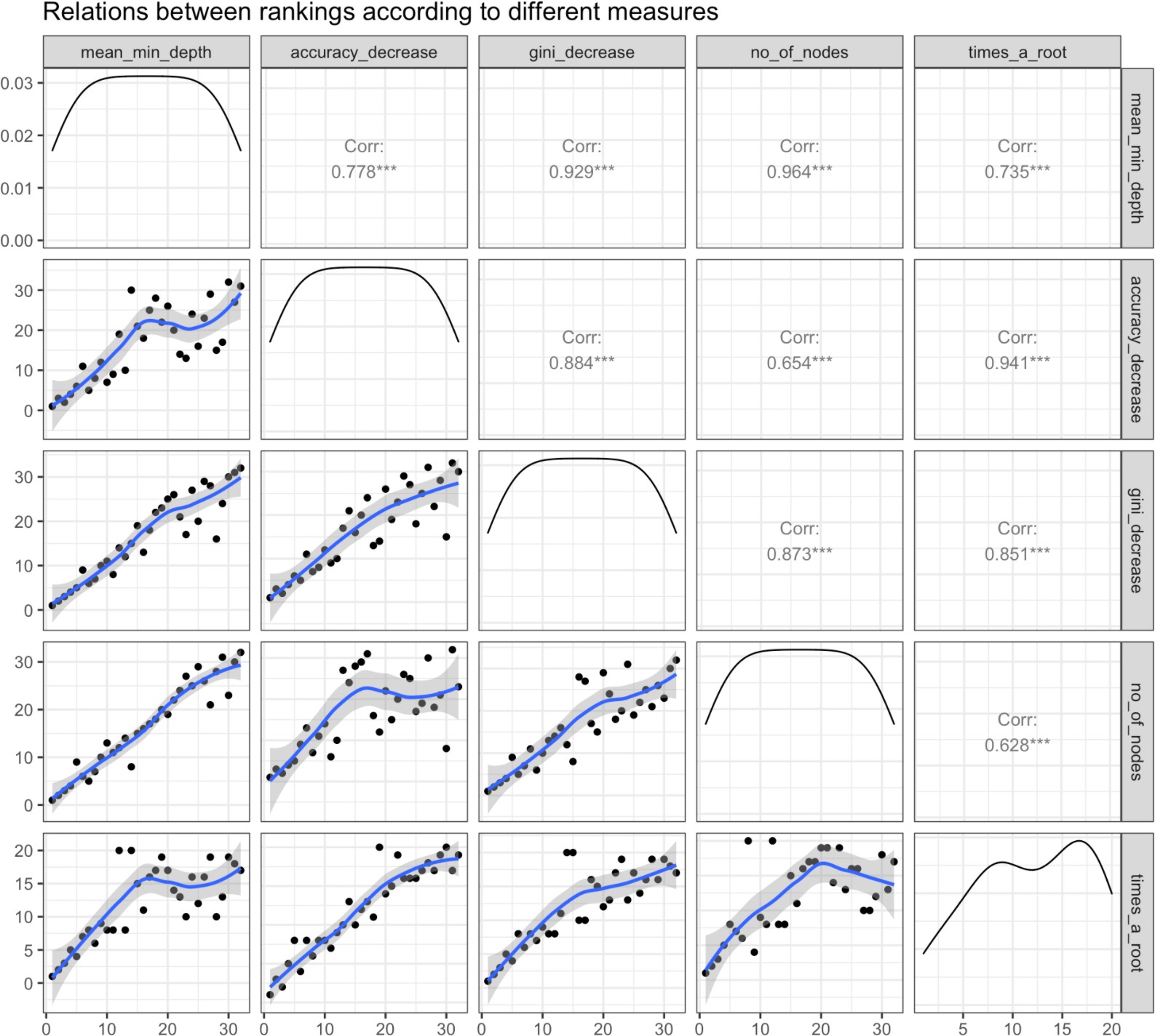
Compare rankings of variables.

The plot below shows bilateral relations between the rankings of variables according to chosen importance measures. This approach might be useful as rankings are more evenly spread than corresponding importance measures. This may also more clearly show where the different measures of importance disagree or agree.

#
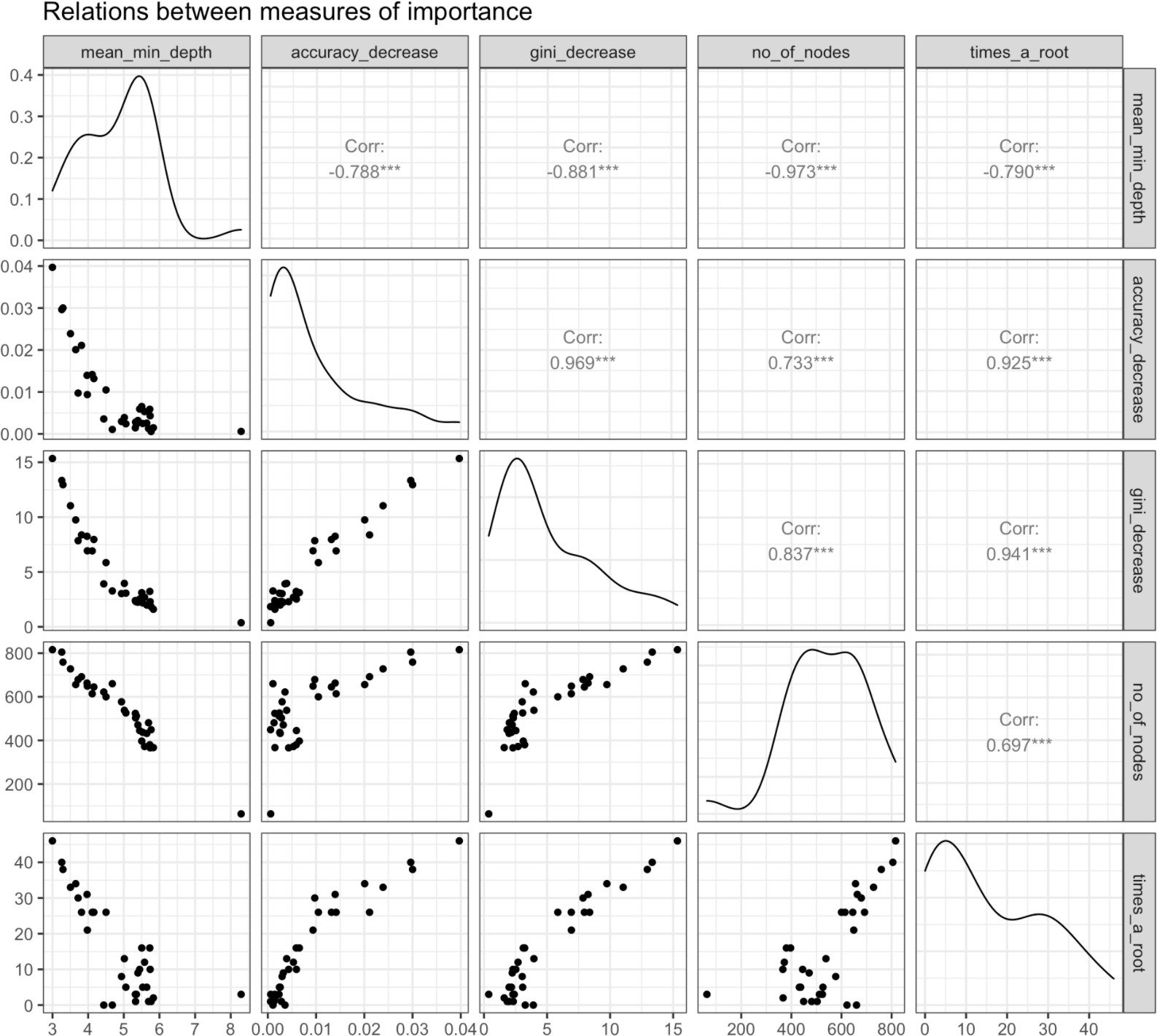
Compare importance measures.

The plot below shows bilateral relations between the following importance measures: mean_min_depth, accuracy_decrease, gini_decrease, no_of_nodes, times_a_root, if some variables are strongly related to each other it may be worth to consider focusing only on one of them.
