# Supplementary material for: Prediction of femoral osteoporosis using machine-learning analysis with radiomics features and abdomen-pelvic CT: A retrospective single center preliminary study
Source: PLoS One. 2021 Mar 4;16(3):e0247330. doi: 10.1371/journal.pone.0247330 (PMC7932154; doi:10.1371/journal.pone.0247330)
Supplement: Attachment — Submitted filename: Point by point response of Reviewer comment_plos One.docx [file pone.0247330.s003.docx]

**Point by point response of Reviewer comment**

**Response to the general comments by editor and editorial office:**

1. We revised the financial disclosure section information.

a. Please provide an amended Competing Interests Statement that explicitly states this commercial funder, along with any other relevant declarations relating to employment, consultancy, patents, products in development, marketed products, etc.

🡺 We provide an amended competing interest statement as following,

“The DongKook Life Science. Co., Ltd. has no competing interests relating to employment, consultancy, patents, products in development, marketed products, etc. These funders had no role in study design, data collection and analysis, decision to publish, or preparation of the manuscript. These do not alter our adherence to PLOS ONE policies on sharing data and materials.”

b. Please include your amended Competing Interests Statement within your cover letter. We will change the online submission form on your behalf.

🡺 We add the amended Competing Interests Statement within our revised cover letter.

2. We include captions of supporting information files at the end of our manuscript.

Supporting information files

S1. Table of five-fold cross validation results of random forest model.

S2. Appendix of graphical explanation of random forest model.

3. We added data availability statement in the cover letter and manuscript.

**Data Availability:** The datasets in this paper can be fully accessed through the following address.

*“Ha, Hong Il; Lim, Hyun Kyung; Park, Sun-Young; & Han, Junhee (2021). Prediction of femoral osteoporosis using machine-learning analysis with radiomics features and abdomen-pelvic CT: A retrospective single center preliminary study. Zenodo. https://doi.org/10.5281/zenodo.4460972”*

Comment and response of Reviewer #1

*1. Did you evaluate the validity of the model by cross-validation? If not, please add it. If you have done so, please add it to the supplementary materials.*

🡺 We performed 5-fold cross validation and added it to supplementary material named as “S1. Table of five-fold cross validation results of random forest model”.

*2. Please indicate the hyperparameters of Random Forest. Did you adjust the hyperparameters in this study? If so, please add the optimized method and result. If you haven't done so, expect to tune for this result.*

🡺 We add the tuning of hyperparameters of random forest model as follows,

“For the best hyperparameter tuning, five-fold cross validation using random search was performed and the result was summarized in supplementary file (S1. Table of five-fold cross validation results of random forest model). The hyperparameter of our RF algorithm were as follows: mtry=3, minimum nodal size=11, and splitrule=extratrees. The best model was selected and validated in the test cohort. Further explanation of RF model and features was summarized in supplementary file (S2. Appendix of graphical explanation of random forest model).

*3. Which data did you use when you took multiple CT scans during the survey period? Also, did any of the same patients have multiple BMD measurements? Please indicate whether the patient's data used either one or both were included in the analysis.*

🡪 No. 500 APCT cases were obtained from 500 non-duplicated patients and all patients had matched BMD test results. To avoid confusion, we add following sentence.

“There were no duplicate patients enrolled.”

*4. In recent years, deep learning has been attracting attention. The authors have shown very good results in machine learning. Make a comparison with deep learning and show your thoughts.*

🡺 We add following paragraph in discussion section.

“Furthermore, we researched the prediction of osteoporosis by applying machine learning analysis using specific radiomics features and HUHA due to the limited number of patients in this study. Recent AI research is moving to deep learning, which is the evolution of machine learning and it helps in making better precision medicine than machine learning. Deep learning is similar to machine learning, but it does not require artificial intervention. However, it requires big data to train the model otherwise it won’t work as expected. If osteoporosis screening can be implemented by deep learning using wide and easily accessible plain radiographs or large number of CT images in the future, great progress can be expected in the prevention and treatment of osteoporosis.”

**Comment and response of Reviewer #2**

*Please use the following studies in your paper and compare the results:*

1) Radiomics for classification of bone mineral loss: A machine learning study

*Rastegar S, Vaziri M, Qasempour Y, Akhash MR, Abdalvand N, Shiri I, Abdollahi H, Zaidi H. Radiomics for classification of bone mineral loss: A machine learning study. Diagn Interv Imaging. 2020 Sep;101(9):599-610. doi: 10.1016/j.diii.2020.01.008. Epub 2020 Feb 4. PMID: 32033913.*

2) Magnetic resonance imaging radiomic feature analysis of radiation-induced femoral head changes in prostate cancer radiotherapy

*Abdollahi H, Mahdavi SR, Shiri I, Mofid B, Bakhshandeh M, Rahmani K. Magnetic resonance imaging radiomic feature analysis of radiation-induced femoral head changes in prostate cancer radiotherapy. J Cancer Res Ther. 2019 Mar;15(Supplement):S11-S19. doi: 10.4103/jcrt.JCRT_172_18. PMID: 30900614.*

🡪 We thoroughly reviewed the papers presented. However, we found that the experimental methods and the subject of the study differed greatly from our study. Therefore, the comparison and discussion between the results of these papers and the results of our research does not seem to fit the subject of this research theme. However, since both of these papers are valuable papers that analyzed femoral structural changes through radiomics analysis, we cite these papers as references to the radiomics analysis description mentioned in the introduction as #14 and #15, respectively.
