# Supplementary material for: Prediction of femoral osteoporosis using machine-learning analysis with radiomics features and abdomen-pelvic CT: A retrospective single center preliminary study
Source: PLoS One. 2021 Mar 4;16(3):e0247330. doi: 10.1371/journal.pone.0247330 (PMC7932154; doi:10.1371/journal.pone.0247330)
Supplement: Attachment — Submitted filename: point by point response of Reviewer comment2 Plos one.docx [file pone.0247330.s004.docx]

**Response to Reviewers**

Reviewer #1: Thank you for giving me this opportunity to re-review your revised manuscript.

I am happy that almost all of the suggested corrections have been made.

Please refer only to the minor comments below.

Thank you for spending so much time for revised manuscript.

1. Did you evaluate the validity of the model by cross-validation? If not, please add it. If you have done so, please add it to the supplementary materials.

The authors explained that they performed cross-validation to fine-tune the hyperparameters.

Cross-validation is generally an analysis performed to ensure generalization.

Is the result of this analysis of the gini coefficient etc. the holdout method?

Please show the results of cross-validation.

In addition, please show me how to split data for cross-validation. Results may vary depending on the proportion of test data.

Additionally, did you change the hyperparameter adjustment from the default value? Also show the changes from the initial values of hyperparameters

🡺 We attach the original results of 5-folds cross validation confusion matrix. Supplementary file named “S1. Table” is summary of this results.

We divided the total 500 cases into 70% of the train set and 30% of the validation set. And hyperparameter tuning was performed while performing 5-fold cross validation on the train set. The test set result was the validation result of RF model using a tuned hyperparameter by 5-fold cross validation.

In addition, we attach the our R-statistical code. This code contains all information of our random forest model about data split, train and test set composition and hyperparameter searching algorithm, etc. This is critical information of this research because we have 2nd phase study related this research. So, we can't publish this "R-code data". However, we already submit our full data set so anyone can test and validate our research.

A. **Five-fold cross validation confusion matrix results**
1) **Fold 1.**

2) **Fold 2**

3) **Fold 3**

4) **fold 4**

5) **Fold 5**

B. **R- code for random forest model**

library(randomForest)

library(MASS)

library(caret)

library(dplyr)

library(caTools)

library(e1071)

library(tidyverse)

library(tictoc)

library(janitor)

library(doSNOW)

library(ranger)

library(BradleyTerry2)

library(randomForestExplainer)

setwd()

getwd()

data <- read.csv(file="data.csv", header=T)

data["Dx"] <- as.factor(data$Dx)

levels(data$Dx)=c("negative","positive")

set.seed(123)

trainIndex <- createDataPartition(data$Dx, p=.7, list=FALSE, times=1)

train = data[trainIndex,]

test = data[-trainIndex,]

print(table(train$Dx) / nrow(train))

print(table(test$Dx) / nrow(test))

\# K-folds

set.seed(123)

cv_folds_lst <- createFolds(train$Dx, k=5, list=FALSE)

set.seed(123)

cv_folds <- createFolds(train$Dx, k=5, list=TRUE)

ranger_tune_grid <- expand.grid(

.mtry = c(2:32),

.splitrule = c("gini","extratrees"),

.min.node.size = c(2:30)

)

fit_ctrl <-trainControl(method = "repeatedcv",

number = 5,

repeats = 5,

index = cv_folds,

summaryFunction=twoClassSummary,

classProbs = TRUE,

verboseIter = TRUE)

set.seed(123)

gc_grid_ranger_model <- train(Dx ~., train,

method = "ranger",

metric = "AUC",

preProcess = c("zv", "center", "scale", "spatialSign"),

tuneGrid = ranger_tune_grid,

#tuneLength = 15,

trControl = fit_ctrl)

gc_grid_ranger_model

\# The final values used for the model were mtry = 10, splitrule = extratrees and min.node.size = 3.

fit_ctrl <- trainControl(method = "adaptive_cv",

number = 5,

repeats = 1,

index = cv_folds,

search = "random",

adaptive = list(min = 3, alpha = 0.05, method = "BT", complete = FALSE),

summaryFunction = twoClassSummary,

classProbs = TRUE,

verboseIter = TRUE)

gc_ranger_model <- train(Dx ~., train,

method = "ranger",

metric = "Sens",

preProcess = c("zv", "center", "scale", "spatialSign"),

trControl = fit_ctrl,

tuneLength = 7)

gc_ranger_model

for (ntrees in c(25, 50, 100, 250, 500, 750, 1000, 2000)){

print(ntrees)

acc_vec <- c()

for (idx in c(1, 2, 3, 4, 5)){

num <- numeric(idx)

#print(idx)

cv_train <- train[cv_folds_lst != idx,]

cv_validation <- train[cv_folds_lst == idx,]

X_validation <- cv_validation[, -33]

y_validation <- cv_validation[, 33]

formula.init <- "Dx ~ ."

formula.init <- as.formula(formula.init)

set.seed(123)

rf.model <- randomForest(formula=formula.init, data=cv_train, proximity=T, ntree=ntrees,mtry = 3

, splitrule="extratrees", min.node.size=11)

rf.predictions <- predict(rf.model,X_validation, type="class")

#print(rf.model)

#print(confusionMatrix(data=rf.predictions, reference =y_validation, positive="No"))

acc_vec <- append(acc_vec,mean(rf.predictions == y_validation))

}

print(acc_vec)

print(mean(acc_vec))

}

for (idx in c(1, 2, 3, 4, 5)){

num <- numeric(idx)

print(idx)

cv_train <- train[cv_folds_lst != idx,]

cv_validation <- train[cv_folds_lst == idx,]

X_validation <- cv_validation[, -33]

y_validation <- cv_validation[, 33]

formula.init <- "Dx ~ ."

formula.init <- as.formula(formula.init)

rf.model <- randomForest(formula=formula.init, data=cv_train, proximity=T,ntree=500,mtry = 3,

splitrule="extratrees", min.node.size=11)

rf.predictions <- predict(rf.model,X_validation, type="class")

print(confusionMatrix(data=rf.predictions, reference =y_validation, positive="positive"))

}

X_test <- test[, -33]

y_test <- test[, 33]

formula.init <- "Dx ~ ."

formula.init <- as.formula(formula.init)

set.seed(123)

rf.model <- randomForest(formula=formula.init, data=train, proximity=T,ntree=500,mtry =3, splitrule="extratrees", min.node.size=11)

rf.predictions <- predict(rf.model,X_test, type="class")

confusion <- confusionMatrix(data=rf.predictions, reference =y_test, positive="?缺")

rf.model$importance

varImpPlot(rf.model)

X_test <- test[, -33]

y_test <- test[, 33]

test_predict <- predict(rf.model,X_test, type="class")

levels(y_test)=c(0,1)

levels(test_predict)=c(0,1)

y_test <- as.numeric(levels(y_test))[y_test]

test_predict <- as.numeric(levels(test_predict))[test_predict]

train_model <- rf.model$predicted

train_model <- as.factor(as.numeric(train_model))

levels(train_model)=c(0,1)

library(pROC)

par(pty="s")

trainROC <- roc(y_train ~ as.numeric(levels(train_model))[train_model],plot=TRUE,print.auc=TRUE,col="blue",

lwd =4,legacy.axes=TRUE,main="ROC Curves", percent=TRUE)

#ylab="False Positive Percentage", xlab = "True Positive Percentage"

```
## Setting levels: control = 0, case = 1
## Setting direction: controls < cases
```

testROC <- roc(y_test ~ test_predict,plot=TRUE,print.auc=TRUE,col="red",lwd = 4,print.auc.y=45,legacy.axes=TRUE,add = TRUE, percent=TRUE)

```
## Setting levels: control = 0, case = 1
## Setting direction: controls < cases
```

legend("bottomright",legend=c("Training set","Test set"),col=c("blue","red"),lwd=4)

library(ROCR)

library(pROC)

library(randomForest)

#data dependent variable set

set.seed(123)

train$Dx = as.factor(train$Dx)

data1.rf <- randomForest(Dx ~., data=train, proximity=T,ntree=500,mtry = 3, splitrule="extratrees", min.node.size=11)

test$Dx = as.factor(test$Dx)

data2.rf <- randomForest(Dx ~., data=test,proximity=T,ntree=500,mtry = 3, splitrule="extratrees", min.node.size=11)

par(pty="s")

set.seed(123)

require(pROC)

rf.roc1 <-roc(train$Dx,data1.rf$votes[,2], plot=TRUE, legacy.axes=TRUE, percent = TRUE,

xlab="1-Specificity",ylab='Sensitivity',col='#FF0000',

lwd=4,

print.auc=TRUE,print.auc.y=25)

rf.roc2 <-roc(test$Dx,data2.rf$votes[,2], plot=TRUE, legacy.axes=TRUE, percent = TRUE,

xlab="1-Specificity",ylab='Sensitivity',col='#0000FF',

lwd=4,

print.auc=TRUE,add=TRUE, print.auc.y=20)

ci(rf.roc1)

ci(rf.roc2)

roc.test(rf.roc1, rf.roc2, method=c("delong", "bootstrap",

"venkatraman", "sensitivity", "specificity"), sensitivity = NULL,

specificity = NULL, alternative = c("two.sided", "less", "greater"),

paired=NULL, reuse.auc=TRUE, boot.n=2000, boot.stratified=TRUE,

ties.method="first", progress=getOption("pROCProgress")$name,

parallel=FALSE)

library(randomForestExplainer)

library(randomForest)

library(tidyverse)

set.seed(123)

forest <- randomForest::randomForest(Dx ~ ., data = data, localImp = TRUE, proximity=T,mtry = 3, splitrule="extratrees", min.node.size=11)

suppressPackageStartupMessages(suppressMessages(suppressWarnings(explain_forest(forest, interactions = TRUE))))
